# Topological determinants of self-sustained activity in a simple model of excitable dynamics on graphs

**DOI:** 10.1038/srep42340

**Published:** 2017-02-10

**Authors:** Christoph Fretter, Annick Lesne, Claus C. Hilgetag, Marc-Thorsten Hütt

**Affiliations:** 1 Department of Life Sciences and Chemistry, Jacobs University Bremen, D-28759 Bremen, Germany; 2 Department of Computational Neuroscience, Universitätsklinikum Hamburg-Eppendorf, D-20246 Hamburg, Germany; 3 LPTMC, CNRS, UMR 7600, UPMC-Paris 6, Sorbonne Universités, 4 place Jussieu, F-75252, Paris, France; 4 Institut de Génétique Moléculaire de Montpellier, UMR 5535 CNRS, 1919 route de Mende, 34293 Montpellier cedex 5, France; Université de Montpellier, 163 rue Auguste Broussonnet, 34090 Montpellier, France; 5 Department of Health Sciences, Boston University, Boston, USA

## Abstract

Simple models of excitable dynamics on graphs are an efficient framework for studying the interplay between network topology and dynamics. This topic is of practical relevance to diverse fields, ranging from neuroscience to engineering. Here we analyze how a single excitation propagates through a random network as a function of the excitation threshold, that is, the relative amount of activity in the neighborhood required for the excitation of a node. We observe that two sharp transitions delineate a region of sustained activity. Using analytical considerations and numerical simulation, we show that these transitions originate from the presence of barriers to propagation and the excitation of topological cycles, respectively, and can be predicted from the network topology. Our findings are interpreted in the context of network reverberations and self-sustained activity in neural systems, which is a question of long-standing interest in computational neuroscience.

The diverse ways in which architectural features of neural networks can facilitate sustained excitable dynamics is a topic of interest in both the theory of complex networks and computational neuroscience. The existence of stable regimes of sustained network activation, for example, is an essential requirement for the representation of functional patterns in complex neural networks, such as the mammalian cerebral cortex. In particular, initial network activations should result in neuronal activation patterns that neither die out too quickly nor rapidly engage the entire network. Without this feature, activation patterns would not be stable, or would lead to a pathological excitation of the whole brain.

A rich and diverse set of investigations has attempted to shed light on the topological prerequisites of self-sustained activity^1^,^2^,^3^,^4^,^5^,^6^,^7^. One mechanism extensively discussed over the last decade is the phenomenon of reentrant excitations^4^,^7^,^8^,^9^,^10^. These reentrant excitations directly couple the cycle content of a graph to properties of self-sustained activity^4^,^7^,^10^. The general phenomenon of cycles serving as dynamical ‘pacemakers’ in the graph is reminiscent of the cores of spiral waves in spatiotemporal pattern formation^5^,^8^,^9^.

Inspired by the importance of self-sustained activity in neuroscience, we here study, using a minimal discrete model of excitable dynamics with a relative excitation threshold, how network topology affects the propagation of excitations through the network. This investigation allows us to develop a mechanistic understanding of the conditions by which a single excitation in a graph amplifies to generate sustained activity. Our contribution with the present paper is two fold:

1. We investigate how a relative excitation threshold (that is, the minimal fraction of excited neighbors for a node to be excited) affects the usage of structural components (e.g., cycles) in producing reentrant dynamics.
2. We observe two sharp transitions delineating a region of self-sustained activity. The first transition point corresponds to the onset of excitation propagation between the input node, where a single excitation is injected, and the most distant nodes considered as an output layer; it is similar to the epidemic threshold in epidemic diseases, as observed for example in the SIR model^11^,^12^,^13^ (note that in epidemic models, this threshold is in the infection probability, rather than in the relative excitation threshold). The second transition point corresponds to the limit of self-sustained activity and can be related to the occurrence of reentrant excitations.

The approach developed in the present paper provides a simple heuristic to predict, for a given graph and a specific input node, the two transition points observed when varying the relative threshold.

## Methods

### Dynamical model

We use a three-state cellular automaton model of excitable dynamics on undirected networks. Each node can be in an susceptible/excitable (*S*), active/excited (*E*) or refractory (*R*) state. The model operates on discrete time and employs the following synchronous update rules: For a node *i* with *k*_*i*_ neighbors, the transition from *S* to *E* occurs, when at least *κk*_*i*_ neighbors are active. The parameter *κ* thus serves as a relative excitation threshold. In such a scenario, low-degree nodes are easier to excite (requiring a smaller number of neighboring excitations) than high-degree nodes. Quantitatively, 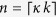 (smallest integer larger than or equal to *κk*) can be considered as the strength with which a node of degree *k* acts as a barrier for propagation, by requiring at least *n* incoming excitations to switch to the excited state.

The model considers only excitatory connections. However, inhibition is implicitly represented in the model due to the automatic transition to a refractory state after excitation. Thus, rather than representing an individual neuron, this model may be thought of as representing a population of coupled excitatory (E) and inhibitory (I) elements as a single node in the network. A node could then for example represent a cortical column consisting of a population of coupled E-I neurons with these populations then linked with each other by excitatory connections.

In neuroscience, there is some evidence that a relative threshold criterion is a plausible activation scenario, as neurons can readjust their excitation threshold according to the input^14^, which typically leads to spike frequency adaptation^15^, and effectively amounts to a relative input threshold. After a time step in the state *E* a node enters the state *R*. The transition from *R* to *S* occurs stochastically with the recovery probability *p*, leading to a geometric distribution of refractory times with an average of 1/*p*. The model (also investigated before^16^) does not allow spontaneous transitions from *S* to *E*, i.e., compared to previous investigations^17^,^18^,^19^, the probability *f* of spontaneous excitations is set to zero. Therefore, the stochasticity of the dynamics is entirely due to the stochastic recovery, controlled by the recovery probability *p*. For *p* = 1, we have a deterministic model, similar to the one discussed in a previous work^5^; there, however, a single neighboring excitation was sufficient to trigger transition to *E*, corresponding to *κ* → 0.

### Details of the numerical experiment

Our numerical experiment starts with a single, randomly chosen input node receiving one excitation, all the nodes being in the susceptible state *S*. We then observe the propagation of excitations (also termed ‘signal propagation’ in what follows) to an output node, selected at random from the nodes at the largest distance from the input node. We either record the excitations accumulated at this output node during a fixed duration *T* (typically *T* = 300 steps, so that the variability of the short transients is averaged out), or observe the absence of propagation reaching the output node. Indeed some of the barriers might not find the required number of active neighbors and fail to propagate the excitation signal. Determinants of successful excitation propagation will thus involve barrier statistics and path multiplicities.

In the present paper, we sample the considered networks in two typical models: random Erdös-Renyi (ER) graphs (generated by wiring *M* edges at random among *N* nodes) and scale-free Barabási-Albert (BA) networks (generated with preferential attachment^20^). For each network, of finite size *N*, we adopt a layered view (as in a previous investigation^16^), according to the shortest distance of the nodes to the input node: the first layer contains the neighbors of the input node, the second layer its second neighbors, and the final layer all the possible output nodes, henceforth termed the output layer. By construction, there are no shortcuts between non-adjacent layers. Using this layered view is motivated by the fact that, due to the refractory period, the excitation signal propagates layer-wise at low enough *κ*, moving forward in a coherent way like a front crossing sequentially each layer. This directionality induced by the dynamics itself should not be confused with an intrinsic directionality of the edges: All the networks considered here are undirected.

Some additional technical comments and side remarks for this section and the following sections are provided in the Supplementary Material.

### Mean-field analysis of excitation propagation

We here adapt a second-order mean-field approach from previous work^16^,^19^ to the present situation estimating the importance of multiple excitations concurring at a given node. The occurrence of such an event at a barrier, i.e. a high-degree node that fails to propagate a single excitation, indeed disrupts our simple prediction topological k^*^ (maximal degree on the easiest path to the output node) of the value 1/*κ*_*c*_ of the onset of excitation propagation. By ‘second-order mean-field approach’, we mean that the computation will use the spatially and statistically average excitation density derived in the mean-field description of the dynamics, but contain a detailed topological analysis of the propagation through a barrier by means of multiple excitations. Only the excitation status of the neighbors of the barrier will be described by the mean-field equations.

#### Mean-field computation of the excitation probability of a barrier

The concept of barrier simply amounts to consider the number 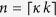 (smallest integer larger than or equal to *κk*) of excited neighbors required for the excitation of this node of degree *k*. It describes the strength with which the node acts as a barrier to excitation propagation. In the layered view we have adopted, what matters for a layer-wise excitation propagation is not only the strength of a barrier but also the number *k*^*in*^ of incoming links from the next upper layer, described through the conditional probability *ρ(k*^*in*^|*k*) given the degree *k* of the barrier. The probability that a node is a barrier of strength *n*, but does *not* act as an obstacle to signal propagation, is thus


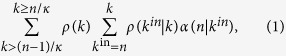


where *α(n*|*k*^*in*^) is the probability to have *n* active nodes among the *k*^*in*^ neighbors of the barrier in the next upper layer. This probability can be computed in a mean-field approximation. Considering that a node get excited if its average number *kc*_*E*_ of active neighbors is larger than *kκ*, leading to the mean-field evolution equations (where *H* is the Heaviside function): *c*_*E*_(*t* + 1) = *c*_*S*_(*t)H*[*c*_*E*_(*t*) − *κ*], together with *c*_*S*_(*t*) = 1 − *c*_*E*_(*t*) − *c*_*R*_(*t*) and *c*_*R*_(*t*) = *c*_*E*_(*t*)/*p*. This yields a steady-state activity density 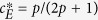 (provided *κ* < *p*/(2*p* + 1))^16^. It comes:


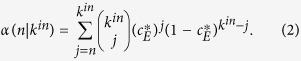


#### Mean-field computation of the probability *P*_*m*_ of multiple excitations

The previous computation is based on the assumption that the propagation is consistent with the layered view, moving forward in a coherent way, like a front crossing as a whole each layer. What then matters to get concurrent excitations is the presence of diamond motifs along the paths. We may alternatively consider that the excitations wander along complicated paths. Indeed, we expect that numerous excitation holes (susceptible nodes failing to get excited) exist for *κ* near *κ*_*c*_, which totally destroys the image of an excitation front propagating layer-wise. In this new view, the network around the barrier is well described by an homogeneous activity density 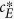, and the excitations could reach a barrier of degree *k* by any of the *k* edges, not only those coming from the next upper layer. This amounts to replace *k*^*in*^ by *k* in Eqs (1) and (2). The probability that a node is a barrier of strength *n*, but does not act as an obstacle to excitation propagation is now simply written 
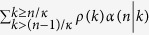
 (it also accounts for the probability that a node is such a barrier). This latter probability has to be summed over all barrier strengths *n* ≥ 2, to get the probability *P*_*m*_ that multiple concurring excitations at barriers allow the excitation signal to propagate up to the output node:


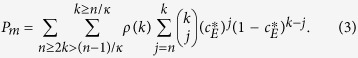


The related expression 1 − *P*_*m*_ will be used to estimate the reliability of our topological prediction k^*^ (largest degree on the easiest path to the output node) of the onset 1/*κ*_*c*_ of excitation propagation, which relies on estimating barrier strengths based on single excitations.

## Results

### Generic properties of the response curve

**Figure 1:**
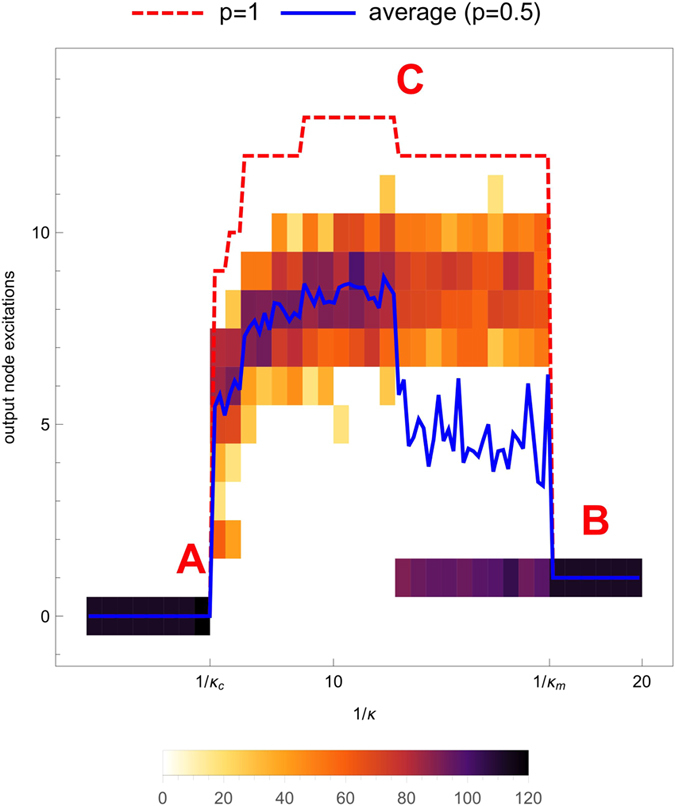
Accumulated output excitations during a fixed duration *T* = 40 steps, for a given ER graph (*N* = 80 nodes; *M* = 320 edges), a single (randomly chosen) input node and a single output node (randomly chosen among the most distant nodes from the input node), as a function of the inverse 1/*κ* of the relative threshold. The plot displays both the deterministic dynamics (*p* = 1, red dashed curve) and the case where the recovery is stochastic (*p* = 0,5; heat map overlay of 30 curves, and as a blue full curve the average over the 30 simulation runs entering the heat map. Initially all nodes were susceptible. Transition points A, B and level reached, C, are indicated, and their prediction in terms of network features is discussed in the corresponding sections below.

The outcome of our numerical experiment produces response curves with very similar features, when plotting the excitation level observed at an output node as a function of 1/*κ*. An example is given in Fig. 1, displaying the three generic features that will be discussed in the following sections: the onset of excitation propagation (point A, value 1/*κ*_*c*_); the limit of self-sustained activity (point B, value 1/*κ*_*m*_) beyond which the excitation signal propagates sequentially through the layers and yields a single record at each output node notwithstanding the duration *T* of the experiment; and the height of the response curve between these two transition points (level C) which increases with the duration *T*.

We chose as a control parameter the inverse of the relative threshold *κ* because 1/*κ* gives the maximal degree a susceptible node can have to be excited by a single excited neighbor. Any node of degree higher than 1/*κ* appears as a barrier, that is, a node for which having a single excited neighbor is not sufficient to be excited. With the choice of 1/*κ* as a control parameter, the transition values can be interpreted in terms of a degree.

The purpose of Fig. 1 is also to show that the transition points of the stochastic model and the deterministic model (i.e. the model with *p* = 1) coincide. When *p* < 1, the stochasticity of the recovery makes possible both propagation failure or non-zero output (for the same network and the same input and output nodes) in the range 1/*κ*_*c*_ < 1/*κ* < 1/*κ*_*m*_, as seen in the heat map in Fig. 1. However, the average over a large enough number of runs (30 runs in Fig. 1) yields an average output curve (blue full curve in Fig. 1) displaying the same sharp transitions as the deterministic response curve (red dashed curve in Fig. 1). In fact the deterministic case delimits the possibility space of the stochastic case: All excitation levels that are possible for the deterministic dynamics are in principle achievable in the stochastic case, if the right nodes have recovered at the right moment.

**Figure 2:**
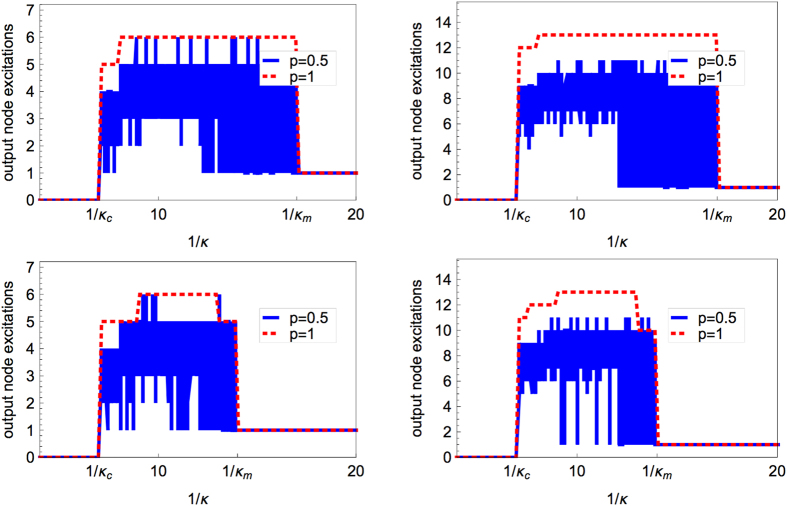
Some examples of response curves for the deterministic (red dashed curve, *p* = 1) and the stochastic dynamics (blue full curve, *p* = 0.5, 30 runs, each displayed as a blue full curve) for an ER graph with *N* = 80, *M* = 320 in the left column and *N* = 80, *M* = 800 in the right column. For each graph, two randomly chosen input nodes (top and bottom panels) have been considered.

We verified that these various features are generic for any ER graph and any choice of input and output node by extensive simulation, and present in Fig. 2 four more examples obtained with other ER graphs. BA graphs behave in a similar way, and this ensemble of networks will be also considered in the systematic and quantitative study of the response curve features presented in the following sections.

#### Prediction of the onset of excitation propagation (point A)

All response curves display a transition value 1/*κ*_*c*_ for the propagation of a single excitation from the input node to the output layer (point A in Fig. 1). This threshold behavior in the absence of spontaneous excitations is analogous to an epidemic threshold: 1/*κ* can be roughly interpreted as a transmission probability. It does not depend on the value of *p*. For a finite network, the transition value 1/*κ*_*c*_ is a random variable depending on the realization of the network, the choice of the input node and an output node in the output layer, and the initial conditions.

For 1/*κ* < 1/*κ*_*c*_ (before point A) the relative number of excited neighbors needed to propagate the excitation signal is so large that each possible path from the input node to the output node contains a barrier stopping the propagating excitation signal before it could reach the output node. A single excitation propagating fails to reach the output node if there exists a node of degree *k* > 1/*κ* along the path. We thus predict the onset of excitation propagation to arise for a value 1/*κ*_*c*_ = k^*^ equal to the smallest (over all linear paths from the input to the output node) of the maximal degree encountered along the path, that is, the largest degree encountered on the easiest path to the output node.

**Figure 3:**
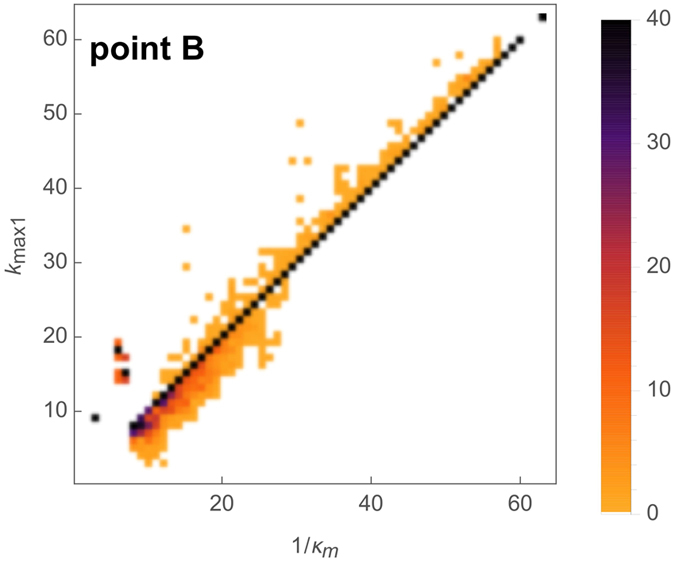
Density histogram of the prediction k^*^ for the onset of excitation propagation (point A in Fig. 1**) as a function of the observed transition value 1/*****κ***_***c***_. The basis of this prediction is that the node with the highest degree on the easiest path (path along which the maximal degree is minimal) from the input node to the output node is limiting, with degree k^*^. Scanning different values of *κ*_*c*_ is obtained by running the dynamics on networks having *N* = 80 nodes and *M* = 100...1900 edges (in steps of 100), considering 10 realizations for each values of *M* and for each network all the possibilities for the input node, while observing the topological quantity k^*^. Data are aggregated into a heat map (color bar on the right, in %) representing the probability density (normalized histogram) of values of k^*^ for each value of 1/*κ*_*c*_.

The quantity k^*^ is determined by considering a randomly chosen node in the output layer. A refined prediction for 1/*κ*_*c*_ is provided by the largest degree k^**^ encountered on the easiest path to the output layer, that is, by considering all linear paths to any node in the output layer. However, the condition becomes less stringent, if the signal propagation activates redundant paths of the same length, so that more than one excitation may spontaneously arrive at a given node. We thus expect that k^*^ and k^**^ would give only an upper bound on 1/*κ*_*c*_, as seen in Fig. 3.

The quality of our prediction is visualized as a scatter plot comparing the topologically predicted transition value k^*^ and the numerically observed value 1/*κ*_*c*_, Fig. 3. In order to compare the latter value, obtained by a binary search algorithm, with the prediction k^*^, we round the numerical result to the nearest integer. Exact matches after rounding are then considered a successful prediction. Note that, as we have observed that the transition value does not depend on the value of the recovery probability *p*, we used *p* = 1 (the deterministic dynamics) to make the binary search reliable.

**Figure 4:**
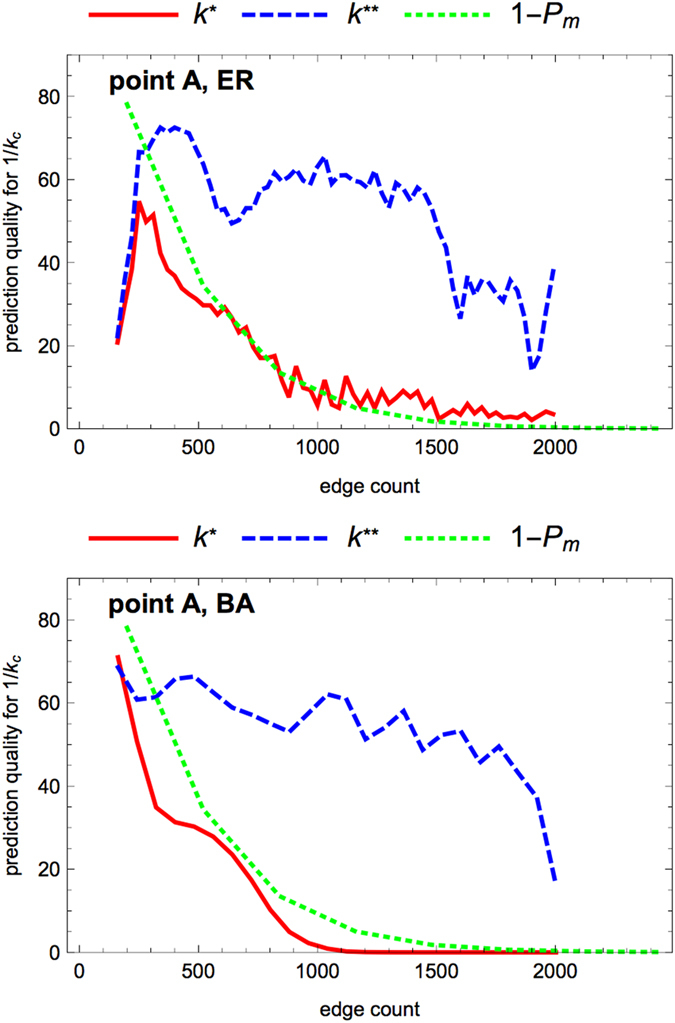
Prediction quality for the onset 1/*κ*_*c*_ of excitation propagation (point A) when increasing the edge density. The data are obtained by scanning graphs having a fixed number *N* = 80 of nodes and a varying number *M* = 150...2000 of edges (steps of 30), considering 50 realizations for each value of *M*, and for each network all the possibilities for the input node. The plot displays the quality of the predictions 1/*κ*_*c*_ = k^*^ (maximal degree on the easiest path to one randomly chosen output node, red full curve) and 1/*κ*_*c*_ = k^**^ (minimum value of k^*^ over all the possible output nodes, blue dashed curve) compared to the numerical value of 1/*κ*_*c*_ (rounded to the nearest integer). Each point in these curves is the number of successful predictions over 4000 trials, normalized so that a perfect prediction would get a score of 100. The dashed green line indicates our confidence in the prediction k^*^, by representing an analytical estimate of the probability 1 − *P*_*m*_ (see Equation (3)) of having no multiple excitations (expected to invalidate the prediction). Upper panel: ER graphs; lower panel: BA graphs.

We computed the quality of our prediction over a large sample of networks and input nodes as the percentage of cases, where 1/*κ*_*c*_ is predicted correctly. In Fig. 4 this prediction quality is shown as a function of the number of edges. As expected, the prediction k^**^ improves the simpler prediction k^*^ in a systematic way. The prediction quality decreases when the graphs become denser. As mentioned above, multiple excitations concurring at a given node, would invalidate our prediction, in allowing this node to be excited even if it were a barrier to the propagation of a single excitation. The probability that a barrier may be passed, because two concurrent excitations reach it, cannot be computed exactly. However we could obtain a mean-field estimate *P*_*m*_, derived in Eq. (3) (see Methods), of the probability of multiple excitations.

As seen on Fig. 1, the critical value 1/*κ*_*c*_ does not depend on the value of *p*. It thus makes sense to look for a purely topological prediction of this value. To check our interpretation that the decrease in quality of our prediction k^*^ is due to concurring excitations alleviating the barrier associated with the node of degree k^*^, we computed the probability of multiple excitations using a mean-field approach. We chose a value *p* = 0.5 of the recovery probability in the range where the mean-field approach gives the most robust and reliable approximation of the actual dynamics. The plot of 1 − *P*_*m*_ (dashed green curve in Fig. 4) demonstrates that the quality of our prediction k^*^ fails in the way we expected when the link density of the graph increases. Curves of 1 − *P*_*m*_ computed for higher values of *p* similarly match the decrease of the prediction quality, supporting our explanation that the failure of our prediction in dense networks originates in the occurrence of multiple excitations (the more frequent the denser the networks).

The plots presented here have been obtained with networks of *N* = 80 nodes. The most interesting phenomenon regarding size dependence is the quality reduction for larger BA graphs due to many hubs distorting the propagating front by imposing dynamical directionality^5^ (see Supplementary Material).

#### Prediction of the limit of sustained activity (point B)

The transition observed in point B is better described when approaching 1/*κ*_*m*_ from above. For large values of 1/*κ*, all nodes have a degree smaller than 1/*κ*. All nodes are thus able to propagate the activity. Starting from the input node, the excitation propagates layer-wise, as a front propagating sequentially across the layers. Each excited layer is followed by a refractory layer, hence no cycling excitations can establish themselves. The only signal amplification comes from the possible branching of paths and an ensuing increase in the number of nodes between one layer and the following one. The single input excitation thus yields a single output excitation per output node, whatever the duration of the observation. Note that the directionality here comes from the dynamics itself (the network is undirected).

When decreasing 1/*κ*, a jump arises in the output signal at some value 1/*κ*_*m*_. Typically a high-degree node, acting as a barrier, is not excited when the excitation front reaches its layer and leaves a susceptible ‘hole’ in a layer of refractory nodes. Such a hole allows an excitation to travel upward, reaching the hole from an excited neighbor belonging the next layer. The surrounding nodes could be no longer in a refractory state, and complex paths are now available for excitation propagation, possibly including cycling excitations if the network is dense enough to contain suitable topological cycles.

At transition point B we observe a strong amplification of excitations. As was discussed previously^5^,^7^,^10^, such an amplification requires the activation of topological cycles in the graph. We suggest that the transition in 1/*κ*_*m*_ can be explained by the appearance of the first active cycle. The amplification observed at point B is sharp. This means that the cycle is traveled several times, or that other cycles can be excited after the first one has stored excitation long enough for some refractory nodes to recover and provide substrate for further cycling excitations.

The analysis of the simulated dynamics in its layered representation for several network realizations shows that as soon as a hole appears in the first layer, other holes rapidly appear in subsequent layers, thus supporting the possibility of numerous cycling excitations, explaining the sharp increase of the output signal (data not shown).

An estimate of the transition point is 1/*κ*_*m*_ = *k*_*max*_, where *k*_*max*_ is the maximal degree encountered in the network. The degree distribution being layer-biased, it can be expected that with a high probability the first hole appears in the first layer. Accordingly, another prediction is 1/*κ*_*m*_ = *k*_*max*,1_, where *k*_*max*,1_ is the maximal degree encountered in the first layer.

**Figure 5:**
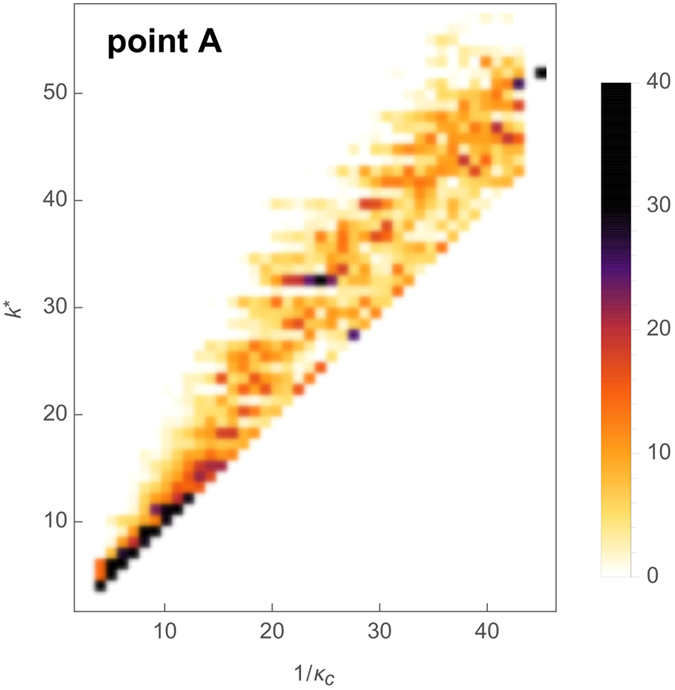
Density histogram of the prediction *k*_*max*,1_ for the limit of sustained activity (transition point B in Fig. 1) as a function of the observed transition value 1/*κ*_*m*_. This prediction corresponds to the maximal degree in the first layer. Scanning different values of *κ*_*m*_ is achieved by running the dynamics on networks having *N* = 80 nodes and *M* = 100...1900 edges (in steps of 100), considering 10 realizations for each values of *M* and for each network all the possibilities for the input node, while observing the topological quantity *k*_*max*,1_. Data are aggregated into a heatmap (color bar on the right, in %) representing the probability density (normalized histogram) of values of *k*_*max*,1_ for each value of 1/*κ*_*m*_.

**Figure 6:**
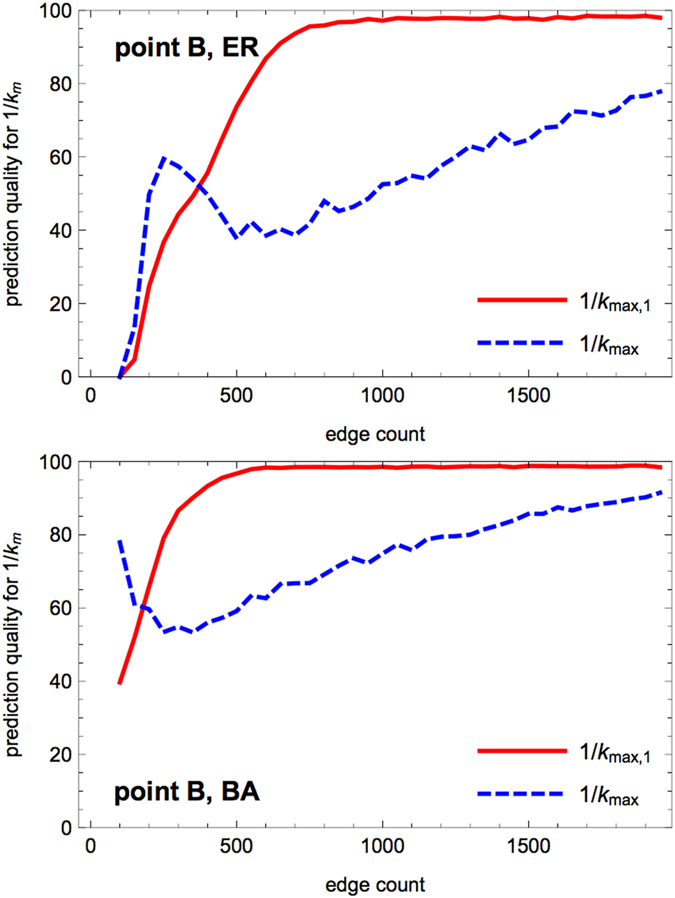
Prediction quality for the limit 1/*κ*_*m*_ of sustained activity (transition point B) when increasing the edge density. The data are obtained by scanning graphs having a fixed number *N* = 80 of nodes and a varying number *M* = 150...2000 of edges (steps of 30), considering 50 realizations for each value of *M*, and for each network all the possibilities for the input node. The plot displays the quality of the predictions 1/*κ*_*m*_ = *k*_*max*_ (maximal degree, blue dashed curve) and 1/*κ*_*m*_ = *k*_*max*,1_ (maximal degree in the first layer, red full curve) compared to the numerical value of 1/*κ*_*m*_ (rounded to the nearest integer). Each point in these curves is the number of successful predictions over 4000 trials, normalized so that a perfect prediction would get a score of 100. Upper panel: ER graphs; lower panel: BA graphs.

We compare our prediction *k*_*max*,1_ with the numerical value observed for 1/*κ*_*m*_ in Fig. 5 (for ER graphs). Again, as we have observed in Figs 1 and 2 that the point B does not depend on the value of the recovery probability *p*, we used *p* = 1 (the deterministic case) to determine 1/*κ*_*m*_. Next, we compare our two predictions 1/*κ*_*m*_ = *k*_*max*_ and 1/*κ*_*m*_ = *k*_*max*,1_ for ER and BA graphs in Fig. 6 as a function of the number of edges.

For dense networks the prediction *k*_*max*,1_ has a 100% quality, meaning that a hole in the first layer is what conditions, directly or indirectly, the onset of a significant amplification of the excitation signal.

In contrast, for sparse graphs, the prediction quality for *k*_*max*_ outperforms the one based on *k*_*max*,1_. When 1/*κ* is small enough for holes to appear in the first layer and be involved in cycling excitation, the signal amplification is already working, due to a hole located in a deeper layer and having a degree *k*_*max*_ > *k*_*max*,1_. Indeed in a sparse graph the occurrence of multiple excitations has a low probability *P*_*m*_ and holes in deep layers are more frequent than in dense graphs.

#### Prediction of the height of the response curve (level C)

From the analysis of the topological determinants of self-sustained activity above, it follows that the activity level at point C in Fig. 1 is linked to the presence of cycling excitations, feeding directly or indirectly the output node. The maximal output excitation level would be reached when the output node is periodically excited due to the activity of a set of cycles, with an average period 2 + 1/*p* prescribed by *p*. This simple argument describing a saturation of the output node yields a prediction of the maximum excitation level *a*_*max*_ at point C depending on *p* and the length *T* of the recording:


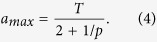


**Figure 7:**
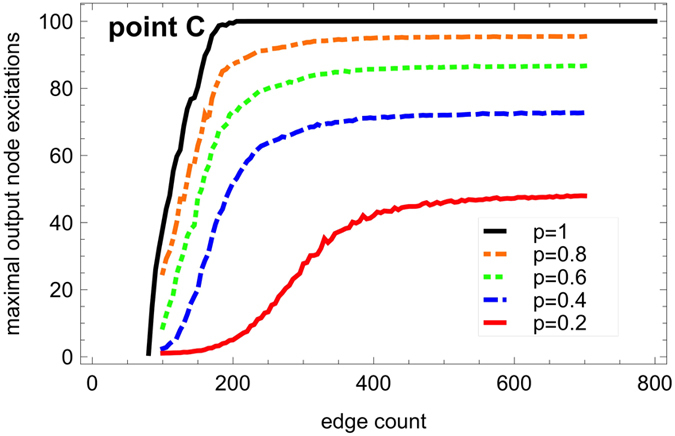
Maximum output signal (level C in Fig. 1) **as a function of the number of edges** ***M***** = 100... 700 (steps of 5) for different values of the recovery probability** ***p***. The number of nodes is constant (*N* = 80). For each value of *M*, 500 network realizations are considered and one input node selected at random. The output excitations are accumulated over *T* = 300 steps, so that the variability of shorter transients is smoothed out. For each network, the maximum value of the output signal is determined by running the dynamics for each value of 1/*κ* between 1 and 50 (step of 1), then the observed maximum is averaged over the 500 network realizations with *M* edges, normalized so that 100 corresponds to the maximal capacity of the output node at *p* = 1. The plot shows that the sustained activity saturates at large *M* (dense graphs) to a value dependent on *p*, with a theoretical maximum value *a*_*max*_|_*p*_ = _1_ = 100 reached for the deterministic dynamics (*p* = 1).

Figure 7 shows the maximal level reached by the response curve for various values of *p* as a function of the edge density. As more and more cycles are wired in the network by added edges, the output node excitation level saturates to a *p*-dependent value, whose maximum is reached for *p* = 1, equal to *a*_*max*_ = 100 for *T* = 300.

**Figure 8:**
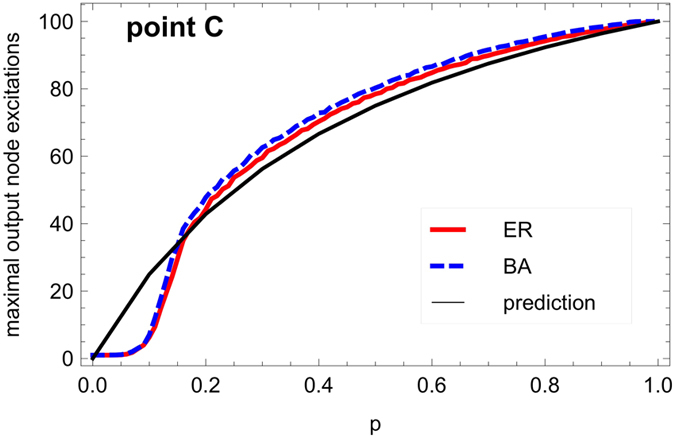
Output excitation saturation level (see Fig. 7 **for details) as a function of the recovery probability** ***p*** **(steps of Δ*****p***** = 0.01), for dense ER and BA graphs (*****N***** = 80,** ***M***** = 640).** ER graphs (red full curve) and BA graphs (dashed blue curve) behave very similarly. The black curve displays the mean-field prediction *a*_*max*_ = 300/(2 + 1/*p*).

The maximal excitation level of the output node as a function of the recovery probability *p* is shown in Fig. 8, for both ER and BA graphs with *N* = 80 nodes and a sufficient number of edges (*M* = 640) to be in the saturation regime (plateaus in Fig. 7). The agreement between the theoretical prediction, Eq. (4) (black line) and the simulation result for *p* > 0.2 supports our explanation in terms of a complex pattern of active cycles ensuring the recurrent excitation of the output node. For *p* < 1, the stochasticity of the recovery could allow re-excitation faster than predicted by the average period 2 + 1/*p*, accounting for the slight excess of the numerical values compared to *a*_*max*_. When *p* = 1, the dynamics is deterministic and sustained activity originating from robust pacemakers becomes possible^5^, such as the triangle *ESR* or the square *ESSR*. The minimum period of 3 is actually observed for *p* = 1, and prediction and simulation perfectly match.

**Figure 9:**
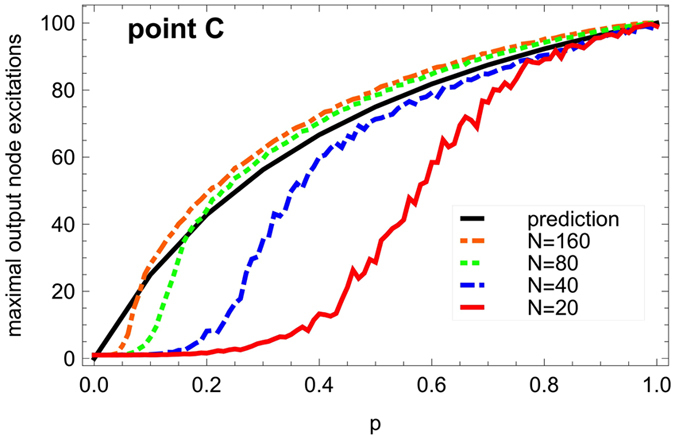
Output excitation saturation level (see Fig. 7 **for details) as a function of the recovery probability** ***p*** **(steps of Δ*****p***** = 0.01, 100 runs for each values of** ***p*****) for dense ER graphs of different sizes** ***N*** **and a large enough number of edges to be located in the saturation plateau in** Fig. 7**, e.g.** ***M***** = 640 for** ***N***** = 80.** The black curve displays the prediction *a*_*max*_ = 300/(2 + 1/*p*).

The marked discrepancy between the prediction and the simulation at low values of *p* is expected since the mean-field-like approximation underlying Eq. (4) is less reliable in the regime of very low excitation densities obtained at low *p*; here the prediction *a*_*max*_ provides only an upper bound on the maximum output excitation level. The kink for small *p* observed in the actual behavior is due to an inability to sustain the activity due to small graph size combined with many refractory nodes. This finite-size effect is further investigated in Fig. 9, confirming its disappearance for larger graphs. This point emphasizes again the importance of studying small or medium-sized graphs, rather than just the asymptotic limit of infinite graphs, as real-world graphs across all domains of application (from biological to social and technological networks) tend to be comparatively small (with numbers of nodes mostly in the hundreds). In small networks, the topological details like the arrangement of cycles and the barrier structure are of importance for qualitative features of the dynamics, while for infinite graphs these details can be expected to average out.

## Discussion

The observed phenomena can be classified as ‘path-driven’ (for the transition in A; *κ* = *κ*_*c*_) or ‘cycle-driven’ (for the transition in B; *κ* = *κ*_*m*_). The onset of excitation propagation in A is due to the appearance of the first barrier-free path. The transition in B between simple signal propagation and signal amplification (onset of sustained activity) is due to topological cycles and the possibility of cycling excitation that occurs as soon as some nodes are not excited in the first stage of signal propagation.

Threshold dynamics have been observed in multiple regions of the brain^21^,^22^. Computational modeling also demonstrated the power of threshold dynamics in predicting empirical neural activity^23^. The present model, which uses a global threshold parameter, in line with similar work^24^, provides a highly simplified representation of threshold mechanisms, which in reality may vary by location as well as in time. However, it is such simplifications that allow the study of the impact of thresholds on network dynamics in the first place, while models that imbue each element with an individual threshold cannot be systematically explored.

Strikingly, the problem of threshold behaviors, which has been investigated in much detail in infectious diseases (e.g., in the SIR model^12^), has not yet been studied extensively for its impact on global neural dynamics. Only a few studies have contributed to a qualitative understanding of the topological and dynamical prerequisites for self-sustained activity^4^,^7^,^10^ (see also the discussion of reentrant excitations in the introduction). While the control of excitation in neural systems may be due to a variety of mechanisms based on cellular properties as well network features^25^, we here focused intentionally on the latter aspect, notwithstanding the existence or importance of cellular mechanisms.

To our knowledge the deterministic cellular automaton model with a relative excitation threshold considered here has not been investigated before. Previously^16^ a stochastic version of this model was discussed. A similar model^24^ emphasizes that such dynamics can serve as a strategy for exploring the relationship of excitable dynamics and network architecture. It has furthermore been studied^16^ how noise facilitates the propagation of excitations (and in particular can lead to an amplification of excitations in a graph). The gradual change of signal propagation with noise intensity is not related to the sharp, topologically determined transition points discussed in our present investigation. The focus of the previous investigation^16^ was to investigate how the interaction of ‘signal’ excitations (excitations correlated with an input signal) with noise (random excitations) enhances – at intermediate noise levels – the signal recorded at the output nodes. While in the previous work^16^ this stochastic resonance phenomenon could be observed numerically, we are still lacking a deep mechanistic understanding of it. An important prerequisite along this way is to understand how the propagation of a single excitation through the network depends on the network’s topological features and the excitation threshold.

In addition to furthering our understanding of the previous numerical observations^16^, we believe that the mechanisms outlined here are instrumental to the onset of self-sustained activity in a network. On a more general level, we here advocate the view that, from the perspective of an input node, an excitation ‘sees’ the network as a pattern of paths and barriers.

The layer representation starting from a given input node provides a node-centered view that a node may have of the network in which it is embedded. This view is relevant in several applications, such as the local probing of a network with no possibility to have an overall and external view, e.g. probing the internet, propagation of signals in neural networks, or social networks in which an individual has only a subjective view of the network.

At intermediate values of 1/*κ*, the excitation dynamics is sensitive to the hierarchical layer representation of the network. In this sense, we have a process-induced layering, which could also happen in real networks, according to a few input nodes have been specifically selected and evolved to match suitable topological features (or a barrier pattern) for the relevant dynamics.

We restrict ourselves to the analysis of comparatively small networks, as many real-world networks (including the cortical area networks, which serve as a source of inspiration for this investigation) are rather small (a few tens up to a few hundreds of nodes). Moreover, the infinite-size limit would give no clue on the impact, investigated here, of specific topological features on activity propagation. In general, we can expect that the differences between network realizations discussed here (i.e. the variation of 1/*κ*_*c*_ and 1/*κ*_*m*_ from network realization to network realization) become much smaller with increasing network size.

Our investigation is inspired by the phenomenon of self-sustained activity in biological neural networks. However, we would like to again emphasize that our results are based on a simple model of excitable dynamics with a relative activation threshold operating on an abstract (random) graph. This minimal setup allowed us to understand some generic mechanisms of how network topology influences the propagation of excitations through a graph and how self-sustained activity sets in. However, in spite of the simplicity of the model, we regard these generic results to be valid in more realistic versions of excitable dynamics and, hence, to be of relevance to collective behaviors in the brain.

The feature of our model that certainly requires some scrutiny is the relative threshold. One mechanism potentially leading to such a relative threshold in the modeling of excitable dynamics is spike threshold adaptation that has been described widely for populations of neurons^26^,^27^,^28^. This phenomenon results in a reduction of the firing rate of highly active neural populations, as resulting from a large number of inputs (i.e., for hub regions). While, admittedly, this adaptation mechanism is not identical to the relative threshold mechanism employed in our paper, it points in the same direction: regions with many inputs may become inhibitory barriers in the path of network activation, at least after a transient phase of high activity.

It should further be noted that the emergence of self-sustained activity has also been addressed using different types of models, for instance, where activation and inhibition are taken into account explicitly^29^.

The transient sustained activity seen in our excitable model is reminiscent of a biological phenomenon termed *network reverberation*, that is, the temporarily sustained activity induced by a specific stimulation of a neural circuit. The concept is related to that of *neural assemblies* introduced by Hebb^30^. One intuitive application of such reverberations may be in dynamic memory circuits, that is, short-term (working) memory based on dynamic patterns. This is in contrast to long-term memory that may be encoded in the synaptic weight distribution of the network. Indeed, one can see transiently sustained activity in specific cortical regions (e.g., prefrontal and posterior parietal cortex) related to working memory tasks, such as a delayed matching-to-sample task. The predominant idea is that reverberations are expressed as *dynamic attractors* of transiently stable increased activity, particularly due to locally increased synaptic strength^31^. This idea provides a link between the dynamic patterns encoding short-term (working) memory and the synaptic weight changes underlying long-term memory. However, there exists an extensive debate on the specific circuitry and parameters underlying the reverberations^32^,^33^,^34^.

## Additional Information

**How to cite this article**: Fretter, C. *et al*. Topological determinants of self-sustained activity in a simple model of excitable dynamics on graphs. *Sci. Rep.* **7**, 42340; doi: 10.1038/srep42340 (2017).

**Publisher's note:** Springer Nature remains neutral with regard to jurisdictional claims in published maps and institutional affiliations.

## Supplementary Material

Supplementary Information
