# Supplementary material for: Topological determinants of self-sustained activity in a simple model of excitable dynamics on graphs
Source: Sci Rep. 2017 Feb 10;7:42340. doi: 10.1038/srep42340 (PMC5301238; doi:10.1038/srep42340)
Supplement: Supplementary Information [file srep42340-s1.pdf]

Supplementary Material to "Topological determinants of self-sustained activity in a simple model of excitable dynamics on graphs"

Christoph Fretter

Department of Life Sciences and Chemistry, Jacobs University Bremen
University Medical Center Hamburg-Eppendorf, Hamburg University

Annick Lesne

LPTMC, CNRS, UMR 7600, UPMC-Paris 6, Sorbonne Universités, 4 place Jussieu, F-75252, Paris, France
Institut de Génétique Moléculaire de Montpellier, UMR 5535 CNRS, 1919 route de Mende, 34293 Montpellier cedex 5, France; Université de Montpellier, 163 rue Auguste Broussonnet, 34090 Montpellier, France

Claus C. Hilgetag

University Medical Center Hamburg-Eppendorf, Hamburg University
Department of Health Sciences, Boston University, Boston, USA

Marc-Thorsten Hütt

Department of Life Sciences and Chemistry, Jacobs University Bremen

1. Additional information

In order to enhance the readability of the main text, we summarize a few technical comments and side remarks in this supplementary information.

1.1. Dynamical model and details of the numerical experiment

Previous investigations have also considered the case of an absolute threshold, where a fixed number (set often to one) of excited neighbors triggers the activation of a susceptible node. They have shown the key role of *hubs* as organizing centers of the activity (Müller-Linow et al., 2008; Hütt and Lesne, 2009). In contrast, in the case of a relative threshold κ considered here, there is no amplification due to a potentially increased excitability of high-degree nodes. For a given node, there is actually a balance between a sufficient number of excited neighbors and the number of susceptible neighbors able to propagate the excitation signal. Overall, the amplification rate at a given node is bounded by $(1-\kappa)/\kappa$.

To check the quality of our predictions, we accumulated the results obtained with several network realizations for each number of edges (10 realizations in Fig. 3 and Fig. 5 of the main text, 50 realizations in Fig. 4 and Fig. 6), then for each network all the possibilities for the input node. For the prediction of $1/\kappa_c$ (Fig. 3 and Fig. 4), we then considered for each input node either a randomly chosen output node (prediction k^*) or all output nodes (prediction k^{**}). For the prediction of $1/\kappa_m$ (Fig. 5 and Fig. 6), we considered for each input node the maximal degree $k_{max,1}$ in the corresponding first layer, and the overall maximal degree k_{max}.

The number of layers depends on the network size and the connectivity (as it can be seen on the extreme case of a fully connected network, displaying only one shell whatever the input node is). In our simulations with networks of 80 nodes, the number of shells typically ranges between 5 and 2.

In the context of the relationships between network architecture and dynamics, considering discrete dynamical models has provided some key insights into the functions of complex networks in the past, e.g. Boolean models for gene regulatory networks (Bornholdt, 2005) and SIR ('susceptible' – 'infected' – 'removed') and SIS ('susceptible' – 'infected' – 'susceptible') models for epidemic diseases in social networks (Pastor-Satorras and Vespignani, 2001).

1.2. Generic properties of the response curve

Due to the definition of the relative threshold κ, the actual transition points $1/\kappa_m$ and $1/\kappa_c$ are expected to take only integer values. The result of the binary search algorithm used to numerically determine these points confirms this expectation, up to a minor amount equal to the precision of the algorithm, and the numerical values will be thus rounded to the nearest integer.

Regarding the comparison of the stochastic model ($p < 1$) and the deterministic model ($p = 1$), in rare situations a higher excitation level is achieved in the stochastic case near transition point B (overshooting of some stochastic curves above the deterministic level in Figure 2). The explanation relies on a mechanism detailed in Sec. V of the main text, according which the persistence of a refractive node makes a 'faster' pacemaker (cycling excitation) accessible.

1.3. Prediction of transition point A

The contribution from multiple excitations does not fully explain the falsely predicted cases for sparse graphs. Therefore, the difference to 100 percent prediction quality in Figure 4 must be due to the more complicated layer structure of sparse graphs. This observation is consistent with the fact that the discrepancy appears for ER graphs, but less so for BA graphs, which has a more stable layer structure due to its hubs.

1.4. Prediction of transition point B

Distinction between k_{max} and $k_{max,1}$. One might think that with increasing density, the node of maximal degree would rapidly come to lie in the first layer. This is not the case, as shown by the discrepancy between the quality curves for the prediction $k_{max,1}$ and for the prediction k_{max}, which lies far below. If the node of maximal degree were always in the first layer, then $k_{max,1} = k_{max}$ and the two curves in Figure 6 would coincide. For dense graphs we observe the presence of a hole in the first layer (of degree $k_{max,1}$) to be important for the onset of signal amplification while the hub of maximal degree (k_{max}) is not so significant. Although a hub would act as a barrier for a single excitation, its location in a deeper layer of the dense graph makes it highly probable that it is reached by concurring excitations and thus propagates the signal without generating a hole (see Sec. IV in the main text; dashed green curve in Figure 4 and Eq. A.3 in the Appendix of the main text).

Sparse graphs vs. dense graphs. What apparently matters most in sparse graphs for signal amplification by recurrent (cycling) excitation is the delayed excitation of a global hub. In contrast, what apparently matters in dense graphs is the delayed excitation of a hub in the first layer. In both cases it is difficult to say whether it is the presence of a hole *per se* which matters, or whether what matters are correlated features (e.g. the presence of a sufficient number of holes, or some more intricate feature of the available cycles). These more complex conditions for the onset of signal amplification are the reason for the low prediction quality of $1/\kappa_m$ in the case of sparse graphs. Improving our predictions would ask for a better understanding of what happens after a hole as appeared in the excitation front. The requirements for recurrent activity in terms of either cycle statistics, paths statistics or simultaneous presence of several holes (i.e. degeneracy of the degree k_{max} or $k_{max,1}$) would deserve further investigations.

1.5. Prediction of height C

In the deterministic case, the output excitation level increases linearly with the duration of the observation T. For $p < 1$, the excitation ultimately vanishes in a finite network; however, for p close enough to 1, a long transient activity is observed, during which the accumulated output signal increases with T. Practically the transient duration grows exponentially with p and is longer than any reasonable simulation length, for example a network with $N = 80, M = 284$ displays a transient duration of order 10^6 time steps around $p = 0.4$ (data not shown).

1.6. Parallel to spiral waves in spatiotemporal pattern formation

The transition from a sequential activation of the layers to self-sustained dynamics involving cycling excitations (transition point B; see Sections III and V) is reminiscent of the formation of spiral waves in spatiotemporal pattern formation (Grace and Hütt, 2015). In contrast to simple propagating (target) waves, spiral waves are autonomously driven by an excitation propagating on a ring serving as the spiral 'core'. In such a scenario of spatiotemporal pattern formation, the circumference of the ring matches the refractory period of the excitable medium, thus forming a minimal spatial structure capable of self-sustained activity.

Such spiral cores are formed for example when a gap in a propagating wave front leads to a curling of the open ends (Geberth and Hütt, 2008; Liao et al., 2011; Garcia et al., 2012), which is quite similar to the hole (not activated node) in one of the layers responsible for transition point B in the present study. Topological cycles in the graph then can play a similar role as the spiral core with the cycle length matching the refractory time (on average $1/p$).

Relatedly, the effect of shortcuts on spiral wave patterns has been studied, both theoretically (Sinha et al., 2007) and experimentally (Tinsley et al., 2005) as another link between self-sustained activity and network topology.

2. Network size dependence

We discussed comparatively small graphs, in order to stay close to the phenomenon of sustained activity in cortical area networks. Thus we did not explore the scaling of the threshold curve with network size. This general topic of scaling with network size would resemble the widely discussed phenomena around the topology-dependence of epidemic thresholds. As a trend, we here observe that our predictions, relying on some localized feature, become worse when the network size increases and all the different dynamical events superimpose (mix up) and the impact of a specific one fades away.

Looking at the system size dependence of our prediction quality, the most interesting phenomenon is the reduction of quality for larger BA graph due to many competing hubs.

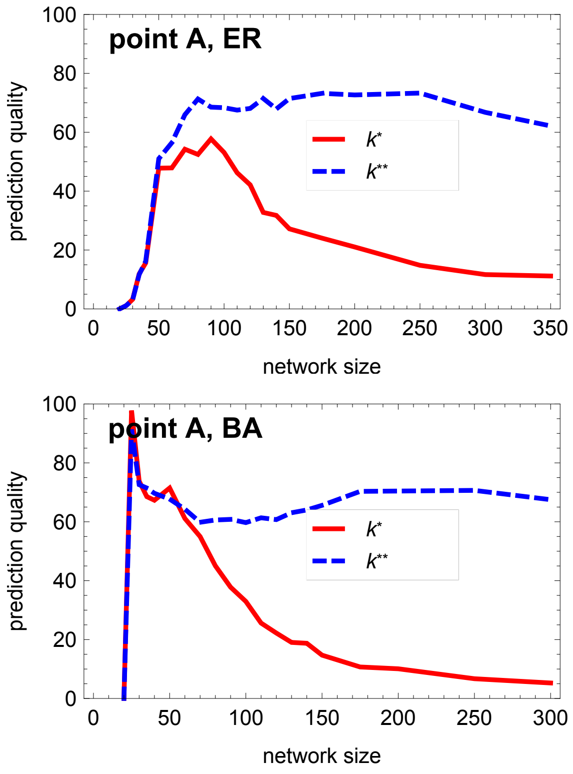

Figure 1. A plot of the prediction quality for the end of sustained activity ($1/\kappa_c$). The data is for graphs of size $N = 20...350$ and $M = 0.04N^2$. The predictions are i) the node with the highest degree on the easiest path (where the maximal degree is minimal) from the input node to the output node is limiting ii) $1/\kappa_c = 2 + 1/p = 3$ (mean field prediction) and iii) $1/\kappa_c = k_{min}$ (ER and BA graphs).

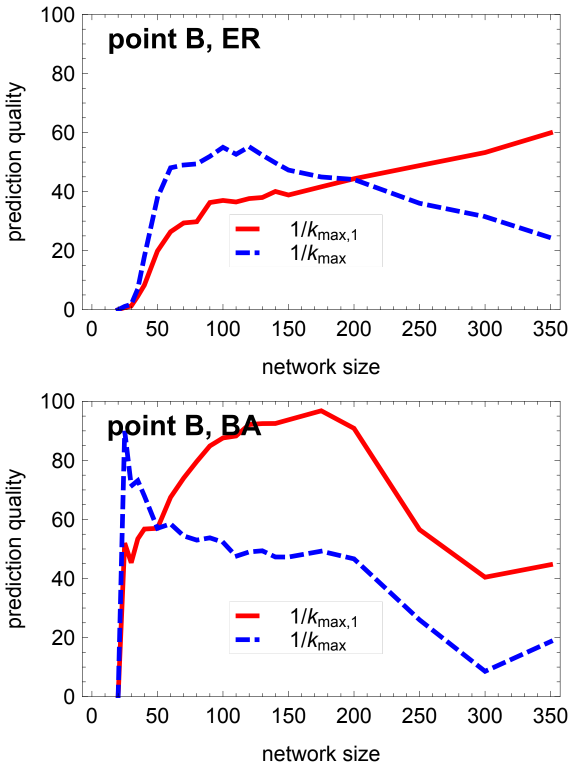

Figure 2. A plot of the prediction quality for the onset of sustained activity ($\frac{1}{\kappa_m}$). The data is for graphs of size $N = 20...350$ and $M = 0.04N^2$. The predictions are that $1/\kappa_m = k_{(max,1)}$ and $1/\kappa_m = k_{max}$ (ER and BA graphs).

References

Bornholdt, S. (2005). Systems biology: less is more in modeling large genetic networks. *Science*, 310(5747):449.

Garcia, G. C., Lesne, A., Hütt, M., and Hilgetag, C. C. (2012). Building blocks of self-sustained activity in a simple deterministic model of excitable neural networks. *Frontiers in Computational Neuroscience*, 6:50.

Geberth, D. and Hütt, M.-T. (2008). Predicting spiral wave patterns from cell properties in a model of biological self-organization. *Physical Review E*, 78(3):1–9.

Grace, M. and Hütt, M.-T. (2015). Regulation of spatiotemporal patterns by biological variability: General principles and applications to dictyostelium discoideum. *PLOS Comput Biol*, 11(11):e1004367.

Hütt, M. and Lesne, A. (2009). Interplay between topology and dynamics in excitation patterns on hierarchical graphs. *Front. Neuroinform.*, 3.

Liao, X., Xia, Q., Qian, Y., Zhang, L., Hu, G., and Mi, Y. (2011). Pattern formation in oscillatory complex networks consisting of excitable nodes. *Physical Review E*, 83(5):056204.

Müller-Linow, M., Hilgetag, C. C., and Hütt, M.-T. (2008). Organization of Excitable Dynamics in Hierarchical Biological Networks. *PLoS Computational Biology*, 4(9):e1000190.

Pastor-Satorras, R. and Vespignani, A. (2001). Epidemic spreading in scale-free networks. *Physical Review Letters*, 86:3200–3203.

Sinha, S., Saramäki, J., and Kaski, K. (2007). Emergence of self-sustained patterns in small-world excitable media. *Physical Review E*, 76(1):015101.

Tinsley, M., Cui, J., Chirila, F., Taylor, A., Zhong, S., and Showalter, K. (2005). Spatiotemporal networks in addressable excitable media. *Physical Review Letters*, 95(3):038306.
